# Parental engagement in an early intervention program for anorexia nervosa: a qualitative study

**DOI:** 10.1007/s00787-025-02827-1

**Published:** 2025-07-31

**Authors:** Thomas Diot, Alexandra Loisel, Marie Rose Moro, Corinne Blanchet

**Affiliations:** 1 Assistance Publique-Hôpitaux de Paris, Cochin Hospital, Maison de Solenn-Maison des Adolescents, 97 Boulevard de Port-Royal, Paris, 75014 France; 2 Inserm U1018, Team DevPsy, CESP, Paris Saclay University, Paris, France; 3 PCPP, Paris Cite University, Boulogne-Billancourt, F-92100 France

**Keywords:** Anorexia nervosa, Day care, Eating disorders, Early medical intervention, Family-centered approach

## Abstract

Anorexia nervosa (AN) is a severe chronic condition requiring early and effective intervention. Family involvement is considered a cornerstone of adolescent treatment, yet the specific dynamics of early parental engagement into care remain poorly understood. This study aimed to explore the processes underlying parental involvement in early care for adolescents with AN, within the context of a family-based day program (EVAFAM) in France. We conducted a qualitative study using semi-structured interviews with 15 parents (7 fathers, 8 mothers) whose adolescents participated in the EVAFAM program between January 2023 and June 2024. We identified three main processes that lead to parental engagement in care: (1) a grieving process, where parents recognized the chronic nature of the illness and their emotional responses to the diagnosis; (2) an active reconstruction process, where parents adapted their attitudes and behaviors to become therapeutic partners; and (3) the relationship to the institutional care framework, which provided both authority and support, while sometimes generating ambivalence. Engagement was supported by increased knowledge and structured interventions but challenged by emotional strain and uncertainty. Notably, early intervention enabled families to shift from denial to active participation, suggesting that engagement is a progressive construction, not an initial precondition. Understanding the trajectories of parental engagement during early intervention highlights key levers for supporting families, including acknowledging emotional transitions, providing practical tools, and reinforcing institutional alliances. These insights can inform tailored interventions and enhance family-centered care for adolescents with AN.

**Supplementary Information:**

The online version contains supplementary material available at 10.1007/s00787-025-02827-1.

## Introduction

Anorexia nervosa (AN) is a severe condition with one of the highest mortality rate among chronic psychiatric disorders [1, 2]. International guidelines recommend early management of these disorders to limit harmful delays in care that negatively affect prognosis [3–6]. However, one of the main difficulties in managing AN is the availability of care networks, with an access delay often exceeding one year [3, 7]. In France, the lack of specialized facilities and the heterogeneity of services across the country lead to the overuse and congestion of specialized centres [8]. The COVID-19 pandemic period contributed to a significant increase in demand for care, inducing long waiting lists in specialized centres [9, 10]. In the last decades, several countries developed early care programs. The Maudsley Hospital program in London was a pioneer in this area, offering early assessment with a specialized team (< 48 h) and the establishment of a personalized care plan, including a family approach as early as possible [11]. This First Episode Rapid Early Intervention for Eating Disorders (FREED) program has proven effective in reducing the duration of untreated illness [12]. Based on this care model, and in response to local demand in France, we developed an outpatient hospital program combining rapid multidisciplinary evaluation with early and intensive family-based approach (EVAFAM) intervention.

Among treatment options, AN-focused family therapy is widely recognized as one of the most effective approaches for children and adolescent with AN [13, 14]. Family factors can indeed play a major role for clinical recovery in this adolescent disorder, but they can also sometimes inadvertently participate in the very perpetuating mechanisms that maintain the illness [15]. Recent studies have examined the experiences of families in caring for their adolescent with AN. A review by Fox [16] of 239 parents who participated in the treatment of their child with AN identified guilt and parental distress as significant feelings during diagnosis, as well as unmet support needs for caregivers [17], especially siblings [18]. This research into parents’ experiences has been carried out at different stages of eating disorders, from recognition to chronic care. However, no study has looked specifically at parents’ experiences during the early management of this disorder, despite the fact that entry into such care presents a crucial stage.

Parental engagement in care can be defined in various ways and should not be confused with related concepts such as presence, participation, or adherence to care. While these elements are often considered prerequisites for engagement [19], they do not fully capture the specifics of engagement. Several authors have described engagement as a multidimensional process involving emotional, cognitive, and behavioral commitment or investment in care [20], which in turn facilitates the transfer of therapeutic tools to the home setting. Clinicians describe parental involvement in anorexia care as emotionally demanding, yet many parents also feel relieved to take an active role in their child’s recovery [21].

We therefore sought to gain a more detailed understanding of the process by which parents engage in care, to help improve our medical intervention. Our objective was to study the processes of parents’ involvement in care, specifically in the context of early management for adolescents with AN in order to identify the effective resources and challenges encountered.

## Methodology

### Methodological approach

We conducted a qualitative study involving the parents of adolescents who attended the EVAFAM program between January 1, 2023, to June 28, 2024. Our chosen methodology is Interpretative Phenomenological Analysis (IPA) [22] an established method in medical qualitative research, designed to best explore the lived experiences of interviewees in a natural setting, by studying their point of view expressed as freely as possible. IPA is based on an iterative, inductive process, from study design to data analysis. The researchers took into account their own potential sources of bias, including knowledge and experience gained from working in adolescent mental health services and conducting research in the realm of adolescent health (see Reflexivity statement for details). This research follows the Consolidated criteria for reporting qualitative research recommendations [23]. It was approved by a French ethical board (CPP Ile-de-France, identifier 2023-A02047-38) and is registered in the Clinical trial registry under the identifier NCT06218472.

### Context

The EVAFAM day-care program is a specialized AN treatment-center located in the Maison des Adolescents, Cochin Hospital, Paris, France. This program is dedicated to adolescents aged 12 to 18 with a first episode of AN, according to DSM-5 criteria [24], with recent onset of symptoms (12 months or less), and new to specialized care. Adolescents are recruited as part of outpatient consultation for eating disorders within our specialized centre. EVAFAM is a family-based program inspired by Family Based Therapy (FBT) approaches that have become common practice, when possible, in treating adolescents with AN. After an initial assessment of each adolescent and parents, six to eight families are included together for 12 weekly sessions, each lasting three hours, alternating between individual sessions with adolescents and multi-family sessions. The therapeutic approach relies on systemic family therapy theory combining individual care and family-based approach with a multidisciplinary team, including psychiatrists, paediatricians, nurses, and dieticians. The individual sessions with adolescents included therapeutic workshops, individual interviews, paediatric and nutritional follow-up, a psychomotor therapy session, a group-meeting with an expert patient, as well as shared therapeutic meals. Siblings are invited to participate in one of the multi-family sessions.

### Recruitment

All parents who participated in the program with their child during this period were individually contacted by a researcher (TD) via e-mail within a month after their participation to arrange an interview. No prior contact was established between the researcher and the participants before this study, which was made clear to them. These interviews were conducted individually at the time and place chosen by the parent, preferably in a quiet space outside of the care setting. Fifteen parents were included in this study (see Table 1 for parents and patients characteristics). All interviews were conducted in person and lasted an average of 56 min (IQR = 48–62). Data collection by purposive sampling continued until theoretical sufficiency was reached, with the data collection process no longer providing major new or relevant insights [25].

**Table 1:** Characteristics of participants included in the study (*n* = 15)

Parent	Age	Mother/father	Adolescent	Adolescent age at program inclusion (years)	Symptoms duration at program inclusion as noticed by parents (months)	Parents’ socioprofessional category *
E1	40–45 yo	Mother	Adolescent 1	16	< 6	(4) Clerical support workers
E2	50–55 yo	Mother	Adolescent 2	17.5	6–12	(4) Clerical support workers
E3	50–55 yo	Father	Adolescent 1	16	< 6	(3) Technicians and associate professionals
E4	40–45 yo	Mother	Adolescent 3	16	< 6	(2) Professionals
E5	50–55 yo	Father	Adolescent 2	17.5	6–12	(4) Clerical support workers
E6	45–50 yo	Mother	Adolescent 4	16	< 6	(2) Professionals
E7	50–55 yo	Father	Adolescent 4	16	< 6	(2) Professionals
E8	50–55 yo	Father	Adolescent 5	12.5	< 6	(4) Clerical support workers
E9	40–45 yo	Mother	Adolescent 6	16.5	6–12	(4) Clerical support workers
E10	40–45 yo	Mother	Adolescent 7	14	< 6	(4) Clerical support workers
E11	40–45 yo	Father	Adolescent 7	14	< 6	(3) Technicians and associate professionals
E12	40–45 yo	Mother	Adolescent 8	14	6–12	(2) Professionals
E13	40–45 yo	Father	Adolescent 8	14	6–12	(2) Professionals
E14	50–55 yo	Father	Adolescent 9	14,5	6–12	(2) Professionals
E15	50–55 yo	Mother	Adolescent 9	14,5	6–12	(2) Professionals

*International Standard Classification of Occupations (ISCO-08) [28]

### Data collection

After a preliminary literature review and team meetings, drawing on our collective clinical experience, we developed a semi-structured interview guide, with particular care to ask open and non-judgmental questions and to allow participants to express themselves freely. An exploratory version was used for three initial interviews then some rephrasing was performed to enhance, without altering its nature (see Table 2 for the final version).

**Table 2:** Interview guide

1	How did you feel about the proposal to enroll in this day hospital program?
2	How did you experience the sessions at the day hospital?
3	Were there any sessions that you found more difficult than others?
4	How do you think your adolescent experienced these sessions? How did she fare during the sessions and between the sessions (at home)?
5	How did things go regarding your adolescent’s eating during the day hospital program?
6	What is your overall take-away from this participation?
7	Have things changed between you and your family after the program?
8	Did the program have an impact on your personal life? (work, friends, etc.)
9	How did you feel about being in a group with families dealing with the same condition for three months?
10	In your opinion, are there any improvements that could be made to the care provided?
11	Is there anything else you would like to add?

While performing interviews, according to IPA guidelines [26], the guide was continuously adapted to each interview situation (free order of questions, rephrasing, the possibility to add prompts and questions), allowing parents to explore aspects that we had not initially anticipated, in order to examine experiential similarities and differences in their relationship with the eating disorder, their participation in care processes, and the impact of the disorder on each family member. All interviews were recorded using a microphone, manually transcribed, and then destroyed.

### Analysis

The data analysis followed an inductive approach based on Smith’s six-stage IPA method [27]: constant comparative techniques were used to analyze the data, by working through each transcript, at first on a factual understanding level, with later analyses generating more latent or conceptual codes. To minimize bias, we used data analysis triangulation (i.e. separate coding and theme identification by three researchers: TD, AL and CB). All interviews were independently analysed and manually coded by two researchers (TD, child psychiatrist, and AL, paediatrician), without the use of automated software tools, then themes were identified and merged together with another researcher not involved in coding (CB, endocrinologist specialized in AN). Full reflexivity statement is available in Appendix 1. Themes were grouped into meta-themes to construct a theoretical model of parental experience.

## Results

**Fig. 1:**
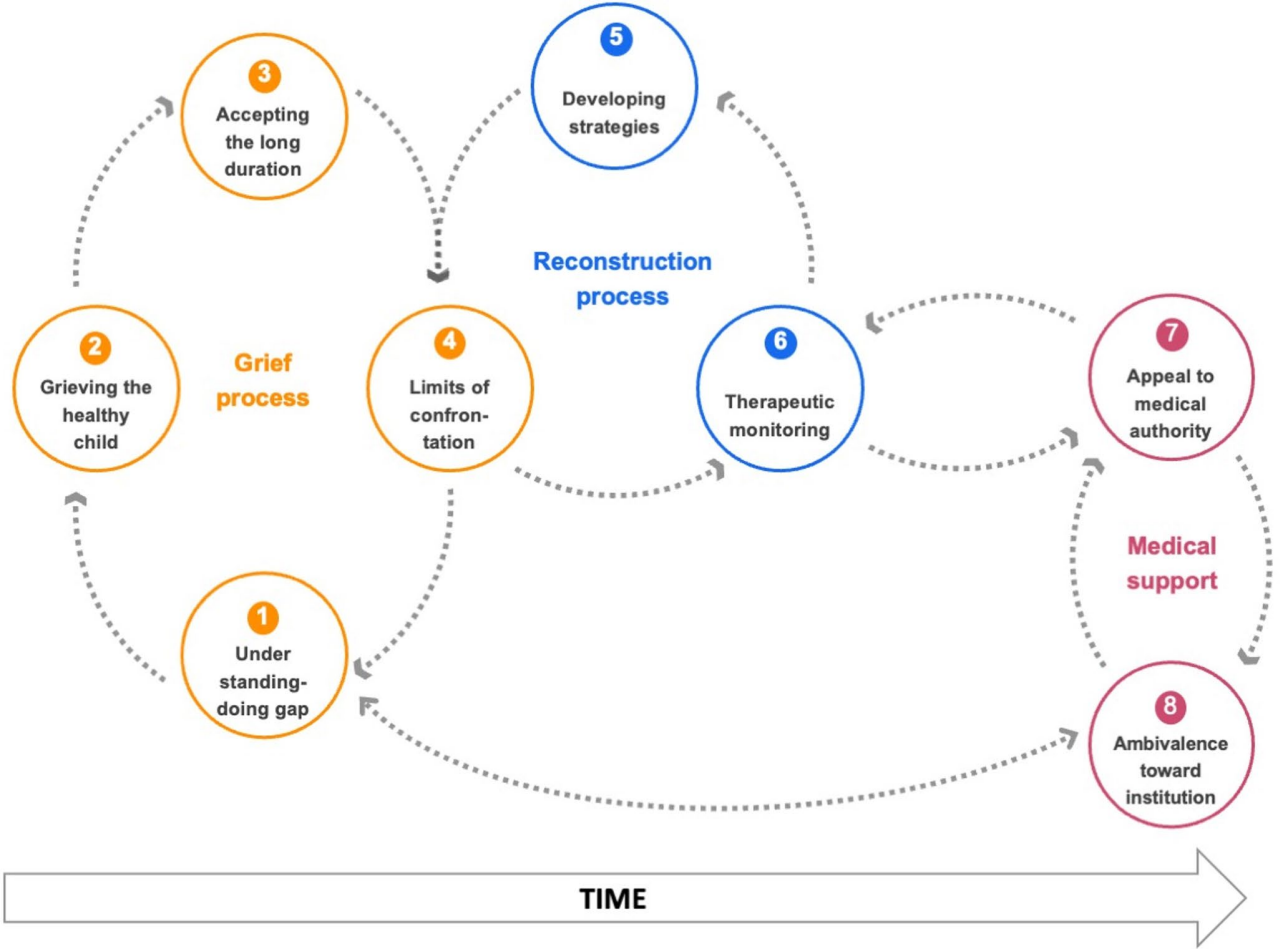
Parental engagement in care takes place through a succession of interconnected and entrenched steps within 3 global processes. First, the parents realize that their child is suffering (1), discover the long-term nature of care (2), increase their knowledge (3) and adapt their way of communicating (4). This process enables them to develop therapeutical strategies (5) that evolve towards an active monitoring role (6). Finally, this process is supported by medical authority (7), although it may be challenged over time (8)

After analysis, three processes can be used to understand the process of parental involvement in care: the grieving process, the active reconstruction process and the relationship to the institutional care framework (Fig. 1).

1. **The recognition and grieving process**
  **Grieving the “healthy” child**
  The period of discovery and recognition of the illness represents a significant upheaval for parents as they come to realize a severe psychological pathology affecting their child, who was previously described as “healthy.” Parents are confronted with a new reality that changes their perception, requiring reassessing many aspects of their child.
  “Because you think you know everything about your child, and then you realize, not at all.” (E3, father)
  In addition to the physical consequences, parents discover unusual or even incomprehensible behaviours in their child, which is very frustrating and painful for them. Thus, parents see an evolution in their adolescent from a “healthy child” to one suffering from a severe, chronic, and paradoxical pathology.
  “She's hit a wall. Is she about to crash into it? I don't know. She's afraid of the hospital, but she makes every effort to get there.” (E3, father)
  **Accepting the long duration**
  Before entering care, the parents' request for care oscillates between despair in the face of the seriousness of the situation and an overestimated hope for rapid recovery. In contrast, some parents, more often men, describe a very passive acceptance of care, and enter this program by default when faced with the seriousness of the situation.
  “At first, it was like, we’ll try to get her out, so she won’t come (to the appointment). If I can get her to gain weight right away, she won’t need to come in three months.” (E3, father)
  Faced with the diagnosis of the illness and the changes it implies, parents develop various adaptation strategies. The first is to accept the chronic nature of care by moving away from concerns about immediate effectiveness and adopting a long-term perspective.
  “When the illness is there, when your kid is surely going to have it for their whole life, when you learn that, damn. She must learn to live with it.” (E8, father)
  This process of discovering and adapting to the illness represents a particularly difficult emotional experience for parents…
  “Sometimes, you leave a part of yourself too. You get caught up in the difficulties your child is going through, and you can’t stay neutral.” (E11, father)
  …which is an opportunity for some to consider personal therapy.
  “You wonder whether you might need therapy for yourself, to think that everyone needs to be treated.” (E11, father)
  Gradually, the parents described a change in their relationship with care, moving from the objective of following this program to a long-term commitment to care.
  “It puts you on the path. After that, it's up to you to take the path. Nobody's going to do it for you.” (E8, father)
  **The understanding-doing gap in managing adolescent behaviour**
  Although many parents shared that they had prior knowledge of the disease, either from relatives or from having suffered from it themselves, we recorded an impressive amount of effort to understand the disease in a very short timeframe. The parents expressed that this improvement in their understanding of the illness was facilitated by the use of group workshops– e.g. role-playing exercises around meals, collective answers to questions raised by the family, or activities facilitating emotional expression - that provide clear, immediately useful information.
  “With all the exercises, it was very rich because we would come out with keys to understand a number of things.” (E5, father)
  However, the numerous parental efforts sometimes result in a discrepancy between their understanding and their practical effectiveness, leaving them feeling somewhat powerless. For example, they sometimes face stark refusal from their adolescent, or cannot strictly apply medical recommendations, which increases their guilt and sometimes leads them to distance themselves from medical advice.
  “There were changes in how we relate to food, in how we relate to her, in how we prepared her meals… But it didn’t result in weight gain.” (E5, father)
2. **The active reconstruction process**
  **Adaptation by recognizing the limits of confrontation**
  Entering treatment coincides with the moment when parents realize and verbalize their own ineffectiveness in helping their adolescent in day to day life.
  “I think we had already understood quite quickly that we had reached our limits in terms of help, that we were no longer contributing much, or on the contrary, it was more conflictual than anything else.” (E5, father)
  Indeed, some parents describe very vertical and violent interactions with their adolescent, aimed at provoking a reaction from the adolescent but which proved ineffective and exhausting. In most cases, a change occurs in the verticality of relationships when parents understand that it is not a simple matter of willpower for their adolescent to eat or not, over which they could exert influence through confrontation, but rather a disorder over which the adolescent does not have complete control.
  “Anyway, confrontation is completely useless, even if sometimes it feels good.” (E5, father)
  **Implementing therapeutical strategies**
  The care program provides an opportunity for discussion and adaptation of family interactions, allowing parents to implement crisis management strategies, such as verbal de-escalation, anticipating anxiety crises, working on conflict avoidance during meals, and focusing on calming conversation topics. In doing so, parents gradually seek a position that allows them to be more authoritative but with less violence, which requires psychological skills.
  “We had to go gently, but at the same time be firm too, because she didn’t want to eat. It takes a huge effort.” (E4, mother)
  In addition to behavioral adjustments aimed at managing crises, parents also described a relational shift within the family, marked by greater emotional openness. The weekly group sessions created a purposed family space, during which intimate matters could be shared. Paradoxically, the public nature of these sessions — and the confrontation with other families and healthcare professionals — facilitated access to the family’s inner world, allowing emotions, bodily sensations, and previously unrecognized family dynamics (often expressed through action rather than words) to be verbalized.
  “And it was very moving, because she opened up — there was really a lot of intimacy that she shared with us.” (E5, father)
  **Transforming parental support into therapeutic monitoring**
  Among the many changes that occur for parents, a marked evolution is noted in the stance they take towards their child and the illness. Initially overwhelmed by their inability to manage the situation and the relational conflict, re-evaluating their role as parents allows them to move towards active, everyday support. Gradually, parents move towards a role of active support and daily monitoring, different from a mere educational parental role.
  “Obviously, there is more vigilance. Observation to detect whether, at any given moment, there is something troubling her that she herself does not see.” (E6, mother)
  Maintaining this delicate balance happens very gradually through a trial-and-error process, with each seeking their appropriate place while being supported by the medical team. Indeed, parents must find a position that allows for active monitoring and support of their child without being too directly coercive while being able to intervene in case of significant worsening of symptoms or crisis.
  “It was necessary to go gently, but on the other hand, firmly too, because she wasn’t willing to eat, so I think it was a bit difficult for my husband.” (E4, mother)
3. **The relationship to the institutional care framework**
  **The medical institution as appeal to authority**
  While parents describe their inability to enforce certain decisions in daily life, they discover the possibility of appealing to medical authority, which helps them overcome this difficulty with better acceptance from the adolescent. Parents learn to rely on this authority by leaning on medical discourse to support their private cues to their adolescent, thus making medical authority an effective third-party partner in the home as well.
  “There was this meal plan that we had pinned onto the fridge. It was like the Ten Commandments, like we were relying on the Tables of the Law.” (E10, mother)
  By sometimes adopting medical discourse and relying on this authority, parents find relief from constant negotiation, and free up family time and energy for topics other than the illness. This third-party partner proves effective for fostering refeeding, but this external intervention can sometimes induce other difficulties for parents, who are then confronted with their lack of personal authority and legitimacy.
  “The ability also to be able to say, ‘It’s what the medical staff said…’ To somehow unburden ourselves at times from this responsibility, which can be guilt-inducing.” (E7, father)
  **Limits of and ambivalence towards institutional support**
  While the medical discourse quickly becomes a support for parents, there are moments of rejection, especially when parents are confronted with failure to implement recommendations. Indeed, this discourse can sometimes be unsettling for the parental role when significant efforts do not yield results.
  “And the doctors would say ‘no.’ You come on Friday, and you look at them, saying,‘You know what, come take my place.’” (E8, father)
  The issue of timing is very present for parents throughout the treatment process, particularly when the program ends, and the transition to monthly outpatient care is discussed. The lack of a clear perspective inherent to this type of condition can be anxiety-inducing for parents.
  “And now what? Because we would have wished it to continue. We reach the end, and we don’t know how much of the road remains.” (E13, father)

## Discussion

The aim of this study was to analyze the process of engagement in care of parents who participated with their adolescents in an early-intervention day care program for AN. We have identified three interconnected processes that lead to engagement in care. We observed parents’ transition into active players in care i.e. parental engagement. Parents also experienced emotional and practical difficulties upon entering care for their child’s AN, including feelings of guilt and grappling with the swift shift from confrontation to a more supportive role. Tensions included shifts in understanding of this complex condition as a chronic mental health issue. Parents expressed feelings of appreciation as the program seemed to improve their understanding, help with family dynamics, bring much valued medical support.

The recommended level of intensity of care in the early stage of AN has been discussed [29]. While inpatient programs are no longer the standard initial approach to AN and outpatient care is initially recommended, most outpatient FBT-inspired approaches require families to actively engage with their adolescents’ symptoms and behaviours, thus being both an outpatient and intensive form of therapy. This implies positioning parents as central partners and even as co-therapists [14, 15, 30, 31]. However, this raises the question of the practical and emotional challenges this represents for families upon entering care, as highlighted in our study by ambivalence in some parents towards several aspects of the program and the gap between understanding and doing that parents expressed. The latest Canadian guidelines for AN recommend entering treatment with a “least intensive treatment environment” [32]. There is indeed a concern about the potential negative effects of prompting adolescents too early to confront their internal conflicts, which could increase the risk of acting out and undermine the psychotherapeutic process [33]. A broader understanding of the different levels and styles of parental engagement in AN care could help better adapt family-centered approaches to each parent’s needs, especially for those who are new to the healthcare system and have little prior experience with the illness.

Based on the parents’ perspective in our study, we can hypothesize that condensing the diagnostic process and initial adjustment to the illness with the therapeutic intervention might alleviate families’ painful experience and strengthen or support parental engagement. However, this can be especially intense and take a toll on parents’ psychological resources. Unlike other studies, no parent described feeling isolated or unsupported. The multifamily aspect of the program and institutional support helped meet the two main parental expectations described in this type of disorder: disease management and individual support [34]. Contrary to other studies [35], we did not observe lesser paternal involvement, nor did fathers voice feelings of exclusion from the care process.

Early care, as observed in this study, seems to rely heavily on the possibility of mobilizing parents as active participants from the outset. Parental engagement may thus represent a key lever in enabling early intervention, particularly when families are able to shift from positions of denial and/or conflict to active involvement. Understanding how this transition occurs could be critical to improving outcomes and tailoring support. It may also help explain why certain families struggle to engage—especially when the adolescent’s denial is mirrored or reinforced by the family system. Identifying the conditions that facilitate or hinder this shift could contribute to more inclusive and responsive care models. These issues have been notably highlighted by Oketah [36], who identified delays in finding help and judgmental attitudes on the part of of professionals.

The long-term impact of such early and intensive interventions regarding clinical stability, risk of reaggravation and long-term treatment outcomes also needs to be studied [37, 38] in order to consolidate and bring further evidence to the “the sooner, the better” intensive approach to AN.

In our study, parents express their involvement in care through significant transformations in their daily strategies, shifting from confrontation to supportive practices, following an initial phase of grief and progressive adaptation, supported by institutional frameworks. This process echoes models observed in other areas of child mental health, such as early intervention in autism spectrum disorder, where parental motivation and readiness to engage in care have been shown to develop gradually as parents reconfigure expectations and construct meaning around the diagnosis and their child’s needs [39].

This study allowed us to unpack various components of parental engagement in the care of adolescents with AN. Although widely recognized as essential in the management of chronic illness—particularly in mental health—parental engagement is not always understood in the same way by healthcare providers and families. For clinicians, engagement often means that parents are able to articulate and share their narrative of the illness, aligning with the medical team’s understanding of the condition. However, for many parents, this expectation may not resonate, especially in the early stages of care. From a social sciences perspective, engagement is traditionally framed as a voluntary act—a form of agency [40]. In the context of AN, however, engagement may feel more like a requirement than a choice, making it a deeply medicalized notion. Faced with this constraint, some parents may attempt to reclaim a sense of agency by “making it their own,” reframing their involvement as a personal and meaningful contribution to recovery. Yet, it remains unclear whether a majority of parents themselves would define their experience in terms of “engagement”. It is also important to consider parents’ personal relationship with food, household food rules, and the limits they set for their children, as emphasized by Loth [41]. Moreover, in the current context of limited healthcare resources, there is a risk that parents may become the primary agents of care for adolescents with AN simply by default, rather than by choice. It is therefore essential to clearly define the scope and limits of their role, to ensure that parental engagement does not turn into a burden or a distorted version of the collaborative involvement we aim to promote.

This study also enabled us to consider some practical implications for healthcare professionals within this program. We found that reference to medical authority and validation constitutes a therapeutic tool particularly valued by parents within this program, contrary to what is sometimes observed in psychiatry, especially when denial of the disorder is significant, or care is coercive [42, 43]. This result further reinforces the legitimacy and self efficacy of teams supporting parents in the care process. Exploring parent’s literacy in AN and especially their cognitive models of the disease and enabling them to enrich their understanding of this complex condition also seems a promising lead. Tailoring approaches to specific family needs and adapting treatment intensity to each patient’s needs are important caveats allowing to better support parents [16, 44, 45].

To complete these findings, future studies could be conducted with healthcare professionals regarding their perception of engagement and early care for both patients and parents.

## Strengths and limitations

This study has several strengths. Firstly, it provides a first-hand account of parental experience of an early intervention program. The interviews were conducted very close to the end of the care program, allowing for feedback that is as close as possible to the participants’ lived reality. This approach minimizes memory bias and reconstructions over time. Secondly, our study includes as many men as women, whereas previous studies were often conducted mainly with mothers [36], we thus provide a more accurate view of family perceptions and dynamics.

The main limitation of this study is the small sample size, due to the limited enrolment capacity of the care program. This may also carry socioeconomic biases. Furthermore, some parents declined to participate due to the clinical worsening of their adolescent or due to time and professional constraints. Lastly, all the adolescents were girls, preventing the study of care experiences among parents of boys with AN.

## Conclusion

By exploring parents' engagement with an early intervention care program for adolescents with AN, this study sheds light on the complexity and evolution of this process in care. Our findings suggest that engagement is not a fixed prerequisite but a dynamic and emotionally demanding process that develops through grief, reconstruction, and institutional support. The early phase of care, while intense, offers a unique opportunity to activate this transformation—especially when parents are supported to move from initial denial to therapeutic involvement. Understanding how engagement is built may be key to improving outcomes, as it allows clinicians to adapt their strategies to each family’s needs and readiness. Further research should examine families for whom this process does not occur, to better understand the barriers to engagement and to design more inclusive interventions. Ultimately, reinforcing the role of families as co-therapists in early stages may represent a pivotal factor in the success of outpatient care for AN.

## Supplementary Information

Below is the link to the electronic supplementary material.

Supplementary Material 1

## Data Availability

The datasets generated during and/or analysed during the current study are available from the corresponding author on reasonable request.
