# Supplementary material for: Parental engagement in an early intervention program for anorexia nervosa: a qualitative study
Source: Eur Child Adolesc Psychiatry. 2025 Jul 31;35(1):167–76. doi: 10.1007/s00787-025-02827-1 (PMC12916978; doi:10.1007/s00787-025-02827-1)
Supplement: Supplementary file 1 — Supplementary Material 1 [file 787_2025_2827_MOESM1_ESM.docx]

**Supplementary material**

#

# Appendix 1 – Reflexivity Statement

Researcher reflexivity is a fundamental aspect of qualitative research. The authors involved in the various stages of this study (design, data collection, transcription, analysis, and writing) considered how their knowledge, personal and professional experiences, as well as their interactions with the research participants, may have influenced the analysis and interpretation of the data collected and took several precautions to limit bias.

The chosen theoretical framework of the study is Interpretative Phenomenological Analysis which was applied upon design, interview guide, data collection, transcription and analysis. This technique has been designed to gain access individuals’ subjective experience, while limiting preexistent bias.

In the research design, and throughout the steps of the research, the authors discussed their perspectives on the topic and designed strategies to limit bias. We subscribe to the COREQ checklist designed to minimize bias and make it apparent when present.

The interview guide was designed by our multidisciplinary team with special care to ask open and non-judgmental questions and to allow participants to express themselves freely.

TD, the author who conducted the interviews and had direct contact with participants, is a child psychiatry resident at the beginning of his research career, trained in qualitative research and Interpretative Phenomenological Analysis. He had no previous knowledge of the program or the patients and families.

We hypothesized that parents’ narratives and their expression of knowledge about the illness could be influenced by the child’s clinical status at the time of the interview. Similarly, we anticipated that the researcher’s medical background could either inhibit or facilitate parents’ verbalization of certain feelings. To address these concerns, the interviewer made it clear from the first contact with participants that he was not clinically involved in the caring team and was not informed of the clinical details of the child involved. He also clarified that participation was anonymous.

The interviews were jointly analyzed by AL, MD, PhD, a pediatrician, specialized in qualitative research and with an initial training in sociology, and CB, endocrinologist, PhD in clinical psychology, and specialist in eating disorders. CB was clinically involved in one session of the program but had no other contact with parents or families by the time of the research. Themes emerged from the data and were not previously identified. Theme identification and selection of meta-themes were carried out in a triangulation perspective by TD, AL and CB. Potential biases that may be linked to authors’ status as well as our efforts to limit them are explained in our methods section.
